# Investigation of the relationship between hot flashes, sweating and sleep quality in perimenopausal and postmenopausal women: the mediating effect of anxiety and depression

**DOI:** 10.1186/s12905-021-01433-y

**Published:** 2021-08-09

**Authors:** Qian Zhou, Baisong Wang, Qi Hua, Qin Jin, Jun Xie, Jing Ma, Furui Jin

**Affiliations:** 1 Menopause Clinic, The International Peace Maternity and Child Health Hospital, Shanghai Jiao Tong University School of Medicine, 1961 Huashan Road, Xuhui District, Shanghai, 200030 China; 2 Department of Statistics, Medical College of Shanghai Jiaotong University, Shanghai, 200030 China

**Keywords:** Menopause, Hot flash, Sleep quality, Anxiety, Depression

## Abstract

**Background:**

To investigate the relationship between sweating from hot flashes, anxiety, depression, and sleep quality in peri- and postmenopausal women. And also the role of anxiety and depression in mediating sweating from hot flashes and sleep quality.

**Methods:**

467 women aged 40–60 years with menopausal problems were enrolled. The sleep quality; hot flashes; sweating; anxiety and depression symptoms were quantitatively evaluated by Pittsburgh Sleep Quality Scale (PSQI), Kupperman Menopause Index, Self-rating Anxiety Scale and Self-rating Depression Scale. Spearman correlation analysis and mediating effect model were used to analyze the relationship between the three.

**Results:**

262 patients’ PSQI score were higher than 6 (58.2%). Total scores of sleep quality were positively correlated with hot flashes, sweating and anxiety and depression symptoms. Anxiety and depression played a mediating role between hot flashes, sweating and sleep quality where the mediating effect of anxiety symptoms accounted for 17.86% (*P* < 0.01) and depression symptoms accounted for 5.36% (*P* < 0.01).

**Conclusions:**

The hot flashes, sweating, anxiety and depression of peri/postmenopausal women are risk factors affecting sleep quality. By alleviating these risk factors, the sleep quality of peri- and postmenopausal women could be improved, which prevents the physical and mental diseases due to long-term severe insomnia.

## Background

Perimenopausal and postmenopausal women usually complain a series of physical and psychological symptoms, called the Menopause Syndrome, resulting from declined ovarian function with fluctuating or decreasing estrogen level. About two-thirds of women suffer from these menopause-related symptoms [1]. And these symptoms include menstrual disorders; vasomotor symptoms (hot flashes and sweating); autonomic nervous dysfunction symptoms (insomnia, palpitations, vertigo, headache and tinnitus); psychiatric symptoms (inability to focus, anxiety, depression, etc.) and genitor-urinary atrophy symptoms.Sleep disorder is common among peri- and postmenopausal women. Long-term severe sleep disorder can seriously affect or damage the physical and mental health of patients. Epidemiological studies have found that insomnia with short sleep duration was associated with increased risk of obesity, hypertension, diabetes, coronary heart disease and other cardiovascular diseases [2–4]. Sleep disorder during menopausal transition period is not only a concomitant symptom of anxiety and depression, but also a risk factor of depression in the future. Therefore, sleep problems among peri- and postmenopausal women should be paid more attention.

There are many factors affecting the sleep quality of peri- and postmenopausal women, such as age, family history of insomnia, previous insomnia episodes, stress, poor health, chronic pain, vasomotor symptoms during menopausal transition period, and neuropsychiatric symptoms which are also strongly related to sleep problems [5–7]. Although peri- and postmenopausal women experience decrease in estrogen levels but not everyone suffers from these symptoms. The pathogenesis of these symptoms is yet not clear, and the causal relationship between these symptoms and poor sleep quality is uncertain. These factors interact with each other to complicate the sleep problems during menopausal transition. Studies have found that hot flashes comprise a specific factor affecting sleep during menopausal transition; the relationship between mood disorders and sleep is bidirectional. 90% of people who have had depression also complained insomnia which is often a complication of anxiety and depression [5]. A study of malignant tumors, rheumatoid arthritis and smoking found that anxiety and depression could mediate pain symptoms, biochemical indicators, drug use, sleep quality and quality of life [8–10]. We speculate that anxiety and depression in menopausal transition may also regulate the relationship between vasomotor symptoms and sleep disorders. Therefore, the purpose of this study was to investigate whether anxiety and depression play a mediating role in hot flashes, sweating and sleep quality. This study provided a new perspective in exploring the relationship between three groups of symptoms, and also scientific evidence in treating sleep disorders among peri- and postmenopausal women.

## Methods

### Inclusion criteria

From March 2016 to February 2017, outpatients from climacteric clinic of International Peace Maternity and Child Health Hospital (IPMCH) were included. The Including criteria were as follows: (1) 40–60 years old; (2) still menstruating with menstrual frequency changes more than twice and/or menstruation period lasting more than 7 days over the last 10 months; (3) at least 1 year after menopause (4) with menopausal syndrome symptoms; (5) conscious and able to fill in the questionnaires. The excluding criteria: (1) Women with primary insomnia before menopause; (2) the woman complains of anxiety or depression, and is taking anti-anxiety drugs or antidepressants.

### Survey conduct and design

This is a hospital-based cross-sectional survey study, using face-to-face questionnaire to survey peri-and postmenopausal women who come to the climacteric clinic for treatment and meet the including criteria.The Pittsburgh Sleep Quality Scale (PSQI), Kupperman Menopausal Index (KMI), Self-rating Anxiety Scale (SAS) SRS.14 and Self-rating Depression Scale (SDS) SRS.11 were used to quantify the sleep quality, hot flashes and sweating symptoms, depressive symptoms and anxiety symptoms of the subjects. These scales were under license, and licenses were obtained each survey.The investigator was fully trained and certified. All the subjects signed the informed consent form. The experiment was approved by Ethics Committee for Medical Research of International Peace Maternity and Child Health Hospital (Ethical approval number:GKLW 2017-104).

The Pittsburgh Sleep Quality Score Scale consists of 19 self-rated items and 5 other-rated items. The 19th self-rated item and the 5 other-rated items were not counted in the scoring. The 18 self-rated items were combined into 7 components, including sleep quality, sleep time, sleep efficiency, sleep disorder, hypnotic drugs and daytime dysfunction. Each component was rated as 0, 1, 2, 3 and was ultimately added together. The higher the score, the poorer the sleep quality. The scale had higher reliability and validity in evaluating sleep quality [11].

The vasomotor symptoms were assessed by the KMI scale which was comprised of 13 indicators. It aimed to evaluate vasomotor symptoms, somatic symptoms, psychiatric symptoms and uro-genital symptoms. Each item has a basic score and a 4-grade degree score (0–3 points). Each item score is equal to the basic score multiplied by the 4-grade degree score and the total KMI score of KMI is the summation of the scores of the 13 indicators.

SAS and SDS are two widely used self-assessment tools to quantify anxiety and depression symptoms. They were easy to comprehend and showed good reliability and validity. The scale consists of 20 items with four-grade scoring system. The anxiety scale has 5 reverse scoring points and the depression scale has 10 reverse scoring points. After the addition of each score, the total rough score was multiplied by 1.25. The integer part was applied as total standard score. The standard score of SAS and SDS was 50. 50–59 represented mild anxiety (depression), 60–69 referred to moderate anxiety (depression), and score higher than 70 meant severe anxiety (depression).

### Statistical methods

SPSS version 24.0 was used for the statistical analysis. Continuous variables were expressed as mean ± standard deviation and categorized variables were expressed as frequency and percentage. Spearman’s rank correlation test was used to analyze the correlation, using a two-sided α = 0.05 as standard. The model of mediation effect was fitted by AMOS 24.0; the mediation effect was tested by Bootstrap method and *P* < 0.05 indicated that the difference was statistically significant.

## Results

### Basic clinicopathological characteristics

A total of 467 women with an average age of 49.68 ± 4.5 years old and an average body mass index of 22.27 5.6 kg/m^2^ were enrolled in this study. They were all menopausal outpatients from International Peace Maternity and Child Health Hospital, including 181 postmenopausal (38.8%) and 286 perimenopausal (61.2%) cases. Of the 467, 125 cases (26.8%) were complicated with hypertension, diabetes mellitus, hyperlipidemia and thyroid diseases. The basic clinicopathological characteristics are shown in Table 1.

**Table 1:** Basic clinicopathological characteristics of the subjects (n = 467)

Characteristics	Number	Percentage (%)
Age		
40–44	57	12.2
45–49	154	40.0
50–54	179	38.3
55–60	57	12.2
Body mass index (BMI)		
< 18.5	11	2.36
18.5–24.9	399	85.4
≥ 25	46	9.85
Marital status		
Married	421	90.1
Single or other	46	9.9
Occupational status		
Employed	394	84.4
Unemployed or retired	73	15.6
Academic qualifications		
Junior high school and below	65	13.9
High school or technical secondary school	151	32.3
University or above	223	47.8
Financial income		
< 400 USD	57	12.2
400–700 USD	125	26.8
700–1500 USD	230	49.3
> 1500 USD	27	5.8
With or without co-morbidity		
Yes	125	26.8
No	342	73.2
Menopausal status		
Yes	181	38.8
No	286	61.2

### The quality of sleep of participants

450 patients completed the sleep quality scale, accounting for 96.4% of all the participants. The overall score of sleep quality was PSQI (6.89 ± 50 p). 262 subjects (58.2%) had PSQI ≥ 6). The details about the 7 components; sleep quality, time of sleep, sleep duration, sleep efficiency, sleep disorder, use of soporific agents and daytime dysfunction are shown in Table 2.

**Table 2:** Characteristics of sleep of the survey subjects

Characteristics	Number	Percentage (%)
Quality of sleep		
Good	92	19.7
Moderate	246	54.7
Poor	97	21.6
Very poor	15	3.3
Sleep time (points)		
0	91	20.2
1–2	233	51.8
3–4	98	21.8
5–6	28	6.2
Duration of sleep		
> 7 h	175	38.9
6–7 h (not including 6 h)	164	36.4
5–6 h (including 6 h)	88	19.6
< 5 h	23	5.1
Sleep efficiency		
> 85%	368	81.8
75–85% (not including 75%)	50	11.1
65–75% (including 75%)	24	5.3
< 65%	8	1.8
Sleep disorder (points)		
0	77	17.1
1–9	153	34.0
10–18	198	44.0
19–27	22	4.9
Use of soporific agents		
None	400	88.9
< 1 time/week	26	5.8
1–2 times/week	15	3.3
≥ 3times/week	9	2.0
Daytime dysfunction (points)		
0	117	26.0
1–2	176	39.1
3–4	117	26.0
5–6	40	8.9
PSQI (6.89 ± 4.37)		
< 6 points	188	41.8
≥ 6 points	262	58.2

### Symptoms of hot flashes, sweating and anxiety and depression

450 subjects completed the Kupperman Menopause Index Scale, Self-rating Anxiety Scale (SAS) and Self-rating Depression Scale (SDS), accounting for 96.4% of the total respondents. The mean ± standard deviation of the three symptoms was 4.21 ± 3.06 for hot flashes, 44.57 ± 6.49 for anxiety and 54.65 ± 12.45 for depression.

### The relationship between sleep quality and hot flashes, anxiety and depression

Spearman correlation analysis showed that the total PSQI score was significantly positively correlated with the symptoms of anxiety and depression in hot flashes and sweating. The correlation coefficients with sweating symptoms of hot flashes were rs = 0.496 (*P* < 0.001), anxiety symptoms were rs = 0.327 (*P* < 0.001), and depression symptoms were rs = 0.265 (*P* < 0.001), which indicated that more severe hot flashes and anxiety and depression symptoms were associated with worse sleep quality.

### Mediating effect of anxiety and depression on hot flashes, sweating and sleep disorders

**Fig. 1:**
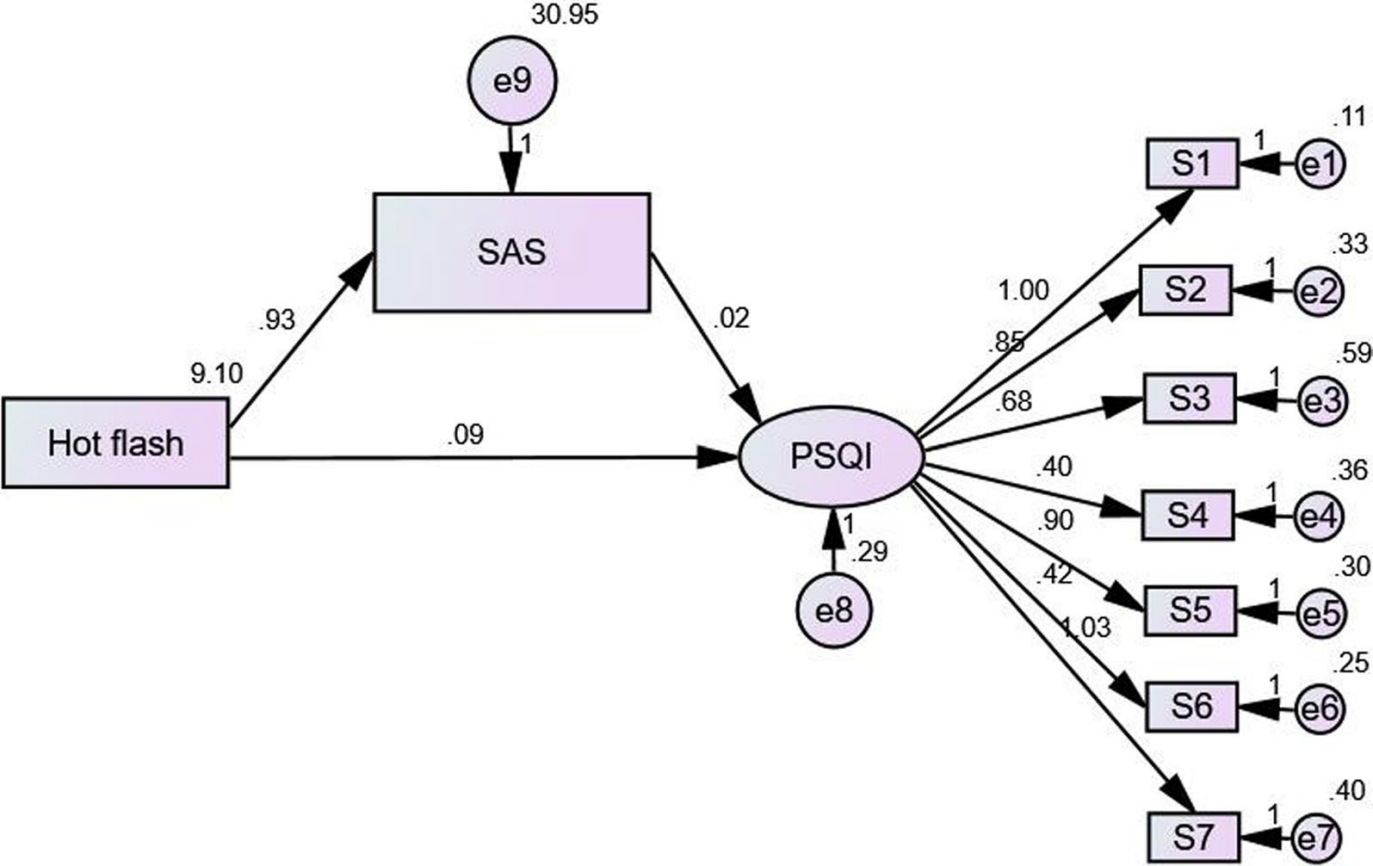
Model of mediating effects between hot flashes and anxiety and PSQI

**Fig. 2:**
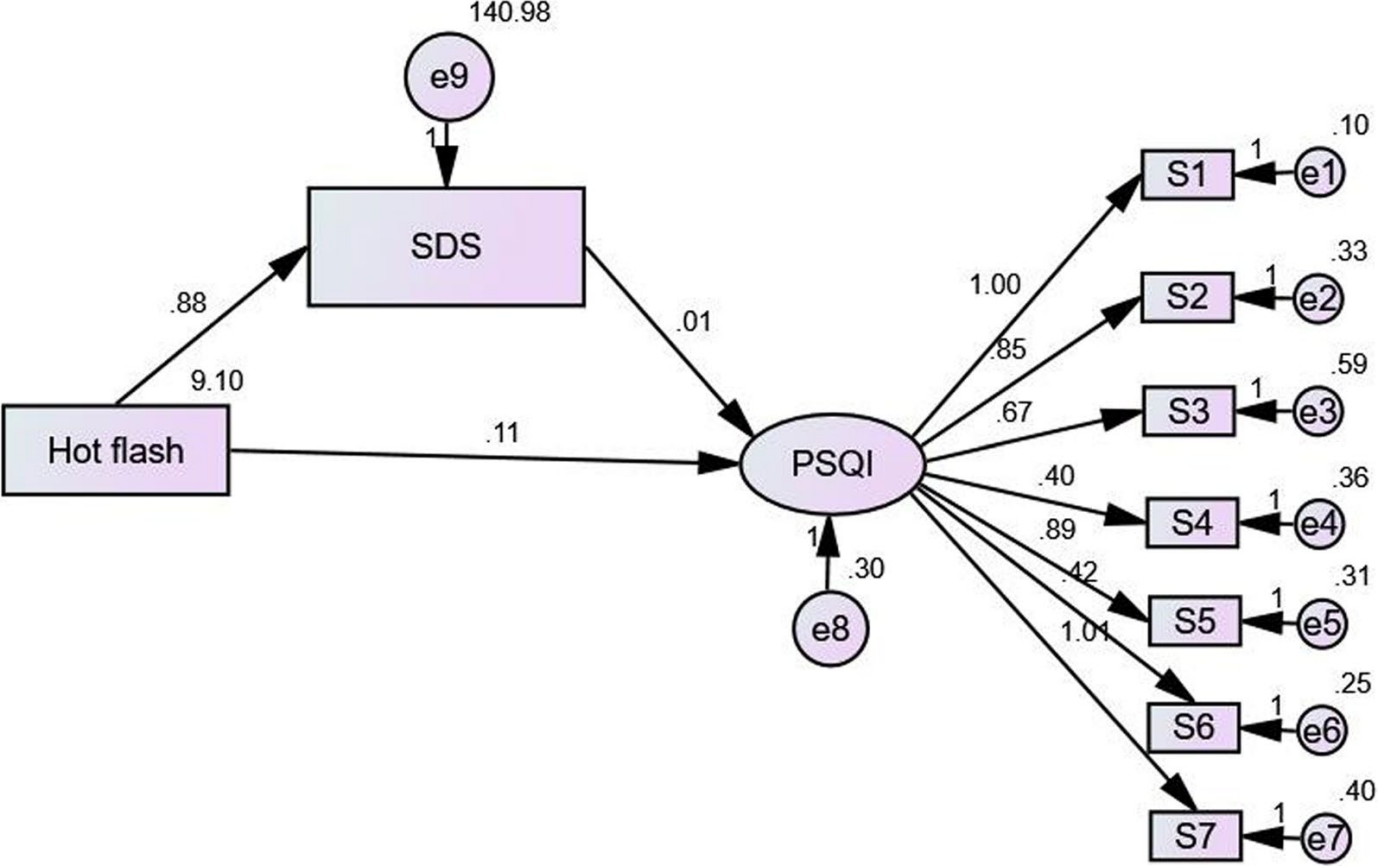
Model of the mediating effects between hot flashes and depression and PSQI

In this study, hot flashes symptom was defined as the observation variable. The independent variable in the model; SDS and SAS were defined as intermediary variable while PSQI as the latent variable. The dependent variable, 7 dimensions of PSQI in the model were defined as observation variable; Hence, the intermediary effect model was constructed. In the model, the SAS fitting index was 2/*df* = 10.084, GFI = 0.888, CFI = 0.829, TLI = 0.763, NFI = 0.815, RFI = 0.744, RMSEA = 0.148. The model fitted well, and the standardization coefficients of each path had statistical significance (*P* < 0.01) (Fig. 1). SDS fitting indexes were: 2/*df* = 9.958, GFI = 0.888, CFI = 0.820, TLI = 0.751, NFI = 0.806, RFI = 0.731, RMSEA = 0.147. The model fitted well, and the standardization coefficients of each path had statistical significance (*P* < 0.01) (Fig. 2).

Bootstrap method was used to test the significance of the mediating effect. The results showed that the direct, indirect and total effects of hot flashes and anxiety on insomnia were statistically significant (*P* < 0.01). The direct effect, intermediate effect and overall effect of hot flashes and depression on insomnia were also statistically significant (*P* < 0.01). Part of the mediating effect between heat sweating and PSQI was established. The indirect effect of hot flashes on sleep disorder was 17.86% through anxiety symptoms (Table 3). The indirect effect of hot flashes on sleep disorder was 5.36% through depression symptoms (Table 4).

**Table 3:** Mediating effect model of hot flash and anxiety and PSQI

Effect	Pathway	Non-standard pathway	Standardized pathway	Standard error	*P* value	95% CI confidence levels	
						Lower	Upper
Direct effect	Hot flashes → PSQI	0.091	0.429	0.012	0.000	0.069	0.116
Intermediate effect	Hot flashes → SAS → PSQI	0.020	0.094	0.006	0.004	0.008	0.033
Overall effect	–	0.112	0.523	0.010	0.000	0.091	0.132

Percentage of intermediate effect: 0.020/0.112 = 17.86%

**Table 4:** Mediating effect model of hot flash and depression and PSQI

Effect	Pathway	Non-standard pathway	Standardized pathway	Standard error	*P* value	95% CI confidence levels	
						Lower	Upper
Direct effect	Hot flashes → PSQI	0.106	0.492	0.011	0.000	0.085	0.127
Intermediate effect	Hot flashes → SDS → PSQI	0.006	0.030	0.002	0.004	0.002	0.004
Overall effect	–	0.112	0.522	0.010	0.000	0.091	0.133

Percentage of intermediate effect: 0.006/0.112 = 5.36%

## Discussion

This study explored and analyzed the relationship between subjective evaluation of sleep quality in peri- and postmenopausal women and symptoms of hot flashes, sweating and anxiety and depression. In this study, 262 subjects (58.2%) had PSQI ≥ 6, which is very consistent with the reported incidence of sleep disorders in perimenopausal women. The epidemiological studies of Terauchi et al. [12] and Xu et al. [13] reported that the incidence of sleep disorders in perimenopausal women ranged from 33 to 51%, which was related to menopausal status and race. The incidence of sleep disorders was higher in Asian women and white women, and lower in Hispanic women. Baker et al. [14] reported that the prevalence of sleep disorders and insomnia in menopausal transition period was 40–60%. Cross-sectional studies by Zhang and Li suggest that the incidence of perimenopausal sleep disorders in Chinese women ranges from 51 to 55% [15, 16]. It can be seen that the incidence of sleep disorder in peri- and postmenopausal women is high and it is not only affecting women's daily functions in the near future, also increasing the risk of cardiovascular disease and depression in the long term. Therefore, sleep problems at this stage deserve much attention.

The results showed that sweating symptoms of hot flashes were positively correlated with sleep quality: Heavy sweating symptoms of hot flashes significantly affected the sleep quality proportionally. Several studies have suggested that hot flashes were associated with sleep disorders and insomnia [17–19]. Freeman et al. [20] showed that hot flashes increased the risk of insomnia, and the adjusted OR value was 1.79 (*P* < 0.001). Vousoura et al. [21] showed that hot flashes significantly increased the risk of frequent waking up at night (OR = 1.85, *P* < 0.001), and decreased the overall sleep quality (OR = 2.00, *P* < 0.001). Freedman et al. [22] studied the mechanism of hot flashes and suggested that hot flashes are the response of the central nervous system to the fluctuation of estrogen. The decrease of estrogen level triggers hot flashes, which are mediated by the activation of the central sympathetic nervous system. The increase of norepinephrine level in the brain and the decrease of combined estrogen level are part of the etiology of hot flashes. The levels of norepinephrine and 5-hydroxytryptamine (5-HT) in the brain are antagonistic. Widely distributed in the brain, 5-HT participates in the regulation of mood, sleep and stress response and is directly related to menopausal sleep disorders whereby the central 5-HT content of insomniacs is relatively lower [23]. It should be noted that the causes of insomnia may be different for women with primary insomnia before menopause and perimenopausal insomnia. The main causes of perimenopause sleep disorders include degradation of body function, decreased estrogen levels, menopausal related symptoms, and stress states, Bad mood and other chronic health problems. However, primary insomnia before menopause are often related to poor mental and psychological conditions and strong cognitive basis [5].

Our results also show that anxiety and depression scores are significantly positively correlated with sleep quality. Zervas et al. [24] suggest that depressive symptoms affect sleep independently of hot flashes. Vousoura et al. [21] showed that depression was associated with difficulty in falling asleep and early awakening (OR = 2.00, *P* < 0.001). The subjects of our study are middle-aged women aged between 40 and 60 years old: With the increase in age, the health status of middle-aged women becomes compromised, sub-health status increases while the incidence of chronic co-morbidities increases. At the same time, work stress and family burden can easily contribute to anxiety and depression. Anxiety and depression cause hyperfunction of hypothalamus–pituitary–adrenal axis (HPA axis) [25, 26], increased sympathetic nerve excitability, increased levels of norepinephrine and cortisol in blood circulation, which make patients in a state of high arousal [4], thus affecting sleep quality. Drug therapy and psychotherapy are the treatment methods for insomnia. The results suggest that psychotherapy or drug therapy can improve anxiety and depression of middle-aged women with obvious anxiety and depression symptoms, and can also help improve their sleep.

The mediating effect model was used to analyze the mediating relationship between anxiety and depression in hot flashes sweating and sleep quality. The results showed that sweating symptoms from hot flashes could affect sleep quality indirectly through anxiety and depression. The effect of anxiety on sleep indirectly accounted for 17.86%, the effect of depression on sleep indirectly accounted for 5.36%, and the influence of anxiety on sleep quality was more significant. Vousoura et al. [21] showed that hot flashes and depression are related to different sleep disorder patterns: the main effect of hot flashes on sleep is frequent awakening during the night, while the main effect of depression on sleep is difficulty in falling asleep and early awakening, but the causal relationship between symptoms is not clear. Our results further suggest that hot flashes can cause insomnia either directly or indirectly through anxiety and depression but the direct effect is more obvious where patients with severe hot flashes may have more significant anxiety and depression. This is consistent with previous research findings: in breast cancer and prostate cancer research, anxiety, depression and fatigue and fatigue play a mediating role in the impact of pain symptoms on patients' quality of life, the overall mediating effect reached 83% [8]; in a study of rheumatoid arthritis, anxiety and depression can mediate the impact of biochemical indicators on patients’ pain [9]. In a study of cocaine dependent smokers, it was found that there was an indirect relationship between sleep quality and drug use results, which was mediated by desire, anxiety and depression. Vasodilation symptoms, emotional disorders and sleep disturbances in peri- and postmenopausal women influence each other, and the interaction is a complex circular relationship, and the causal relationship is uncertain. The relationship between sleep disorders and mood disorders is two-way. Sleep disorders are a predictor of the future development of depression, and depressive symptoms put middle-aged women at risk of sleep disorders [22]. Studies have also found that in women with hot flashes, anxiety may mediate poor sleep[23].Although our research cannot reveal the causal relationship between them, the mediating effect model provides a new perspective for the study of causality, and also provides scientific evidence to improve sleep quality. By relieving the symptoms of hot flashes and sweating and improving the mood of anxiety and depression in middle-aged women through psychotherapy or drug treatment, sleep quality can be improved and long-term cardiovascular disease and depression and other serious physical and mental diseases can be prevented.

There are some limitations in this study. First, this is a cross-sectional study, which has limitations in confirming the causal relationship between the three. Longitudinal cohort research can be designed in the future to further reveal the relationship between the three factors. Second, the sleep quality in this study is self-reported subjective parameter, which may be biased. In the future, a variety of methods, especially objective evaluation of sleep quality can be used for more scientific, accurate and unbiased evaluation.

## Conclusions

This study was the first time to investigate and determine the mediating effect of anxiety and depression between hot flashes, sweating symptoms and sleep quality. This discovery helps to understand the relationship between sleep quality, vasomotor symptoms, anxiety and depression, and provides a new perspective and theoretical basis for improving the sleep quality of perimenopausal and postmenopausal women.

## Data Availability

The datasets used and/or analysed during the current study are available from the corresponding author on reasonable request.

## Abbreviations

PSQI: Pittsburgh Sleep Quality Scale
KMI: Kupperman Menopausal Index
SAS: Self-rating Anxiety Scale
SDS: Self-rating Depression Scale
5-HT: 5-Hydroxytryptamine
HPA: Hypothalamus–pituitary–adrenal axis
